# Activated protein C resistance in the copresence of emicizumab and activated prothrombin complex concentrates

**DOI:** 10.1016/j.rpth.2024.102479

**Published:** 2024-06-18

**Authors:** Yuto Nakajima, Mitsumasa Osuna, Kuniyoshi Mizumachi, Naruto Shimonishi, Shoko Furukawa, Kenichi Ogiwara, Keiji Nogami

**Affiliations:** 1 Department of Pediatrics, Nara Medical University, Kashihara, Nara, Japan; 2 Advanced Medical Science of Thrombosis and Hemostasis, Nara Medical University, Kashihara, Nara, Japan; 3 The Course of Thrombosis and Hemostasis Molecular Pathology, Nara Medical University, Kashihara, Nara, Japan

**Keywords:** antibodies, factor VIII, hemophilia A, protein C, thrombosis

## Abstract

**Background:**

Venous thromboembolic events have been reported in persons with hemophilia A who received emicizumab and activated prothrombin complex concentrate (APCC) concomitantly, but the relevant mechanism(s) remains unclear. We speculated that activated protein C (APC) and antithrombin (AT) resistance might be associated with these adverse events.

**Objectives:**

To investigate APC and AT resistance in factor (F)VIII–deficient (FVIIIdef) plasma in the presence of emicizumab and APCC.

**Methods:**

In pooled normal plasma or FVIIIdef plasma samples mixed with emicizumab (50 μg/mL) and FVIII-bypassing agents, including recombinant FVIIa (2.2 μg/mL), APCC (1.3 IU/mL), or plasma-derived FVIIa/FX (1.5 μg/mL), the suppression effect of AT (0-2.4 μM) and APC (0-16 nM) was assessed by tissue factor–triggered thrombin generation assay. The APC effects in FVIIIdef plasma with the copresence of emicizumab, FII (1.3 μM), and/or FIXa (280 pM) were also examined.

**Results:**

The AT resistance in emicizumab and each bypassing agent was not observed. Moreover, APC dose-dependent suppression effect was observed in pooled normal plasma or FVIIIdef plasma mixed with emicizumab and recombinant FVIIa or plasma-derived FVIIa/FX. However, APC-catalyzed inactivation had little effect on thrombin generation assay potential in FVIIIdef plasma spiked with emicizumab and APCC. The addition of FIXa to emicizumab in FVIIIdef plasma could lead to partial APC resistance. Furthermore, FVIIIdef plasma spiked with emicizumab, FIXa, and FII was markedly resistant to APC-mediated inactivation.

**Conclusion:**

FII and FIXa in APCCs were key clotting factors for APC resistance in FVIIIdef plasma supplemented with emicizumab and APCCs. The APC resistance in persons with hemophilia A receiving emicizumab and APCC may contribute to venous thromboembolic events.

## Essentials

• Activated prothrombin complex concentrates (APCCs) and emicizumab cause venous thrombosis.
• The mechanism(s) by which the combination of emicizumab and APCC causes venous thrombosis remains unclear.
• Mixtures of emicizumab and APCC were resistant to inactivation by activated protein C.
• Factor (F)II and FIXa contained in APCCs were the key factors in this activated protein C resistance.

## 1 Introduction

Venous thromboembolic events (VTEs) are occasionally reported in persons with hemophilia A and hemophilia B [[1], [2], [3]]. The most frequent risk for VTEs appears to be in persons with hemophilia A with inhibitors after the administration of activated prothrombin complex concentrates (APCCs) or recombinant activated factor (F)VII (rFVIIa). Treatment with APCCs for surgery in persons with hemophilia B has also been reported to be a major risk factor for VTEs [4]. In this context, earlier reports suggested that APCCs and rFVIIa therapy in persons with hemophilia with inhibitors were potentially thrombogenic and were associated with thrombotic adverse events such as myocardial infarction, stroke, and disseminated intravascular coagulation [5,6]. These findings have especially highlighted the need for careful evaluation of persons with hemophilia A with inhibitors receiving APCCs or rFVIIa.

Protein C is a key clotting factor in anticoagulant mechanisms. Activated protein C (APC) plays a crucial role in natural anticoagulant mechanisms by moderating the activity of FVa and FVIIIa [7,8]. In addition, antithrombin (AT) is a serine protease inhibitor that inactivates thrombin and FXa and, less efficiently, FIXa, FXIa, FXIIa, and other procoagulant factors [9]. AT functions as a major physiological anticoagulant, accounting for the inactivation of approximately 70% of total thrombin in plasma [10]. APC resistance (APCR) is known to be a major risk factor for VTEs, especially in patients with the genetic defect FV-Arg506Gln (FV Leiden). In these instances, APC fails to cleave Arg^506^ efficiently, resulting in thrombophilia [11,12]. Moreover, APCR has been reported as a risk factor for venous thrombosis and for cerebral ischemic disease in other inherited and acquired coagulation disorders [11]. Regarding AT deficiency, a previous report suggested that AT reduction increased coagulation potentials in plasmas spiked with bypassing agents (BPAs) of persons with hemophilia [13]. AT deficiency and AT resistance (ATR) are also recognized as inherited risk factors of VTEs, and appear to confer the highest clinical risk for hereditary thrombophilia [5,[14], [15], [16]].

Emicizumab is a recombinant, humanized, therapeutic bispecific monoclonal antibody that binds to FIX/FIXa and FX/FXa and mimics FVIIIa cofactor function in the intrinsic tenase complex [17,18]. Emicizumab prophylaxis significantly reduces bleeding episodes in persons with severe hemophilia A, regardless of the presence of inhibitors [[19], [20], [21], [22], [23], [24], [25]]. In a phase 3 clinical trial, the HAVEN 1 study, identified thrombotic events were in 5 emicizumab-treated persons with hemophilia A with inhibitors who had received concomitant therapy with a repeated infusion of APCC [21]. Subsequently therefore, clinical guidelines recommend the avoidance of APCCs during emicizumab prophylaxis and propose the use of rFVIIa (≤90 μg/kg) or the lowest dose of APCC (≤50 IU/kg) for the treatment of breakthrough bleeds in these patients [[26], [27], [28]]. Detailed mechanism(s) responsible for thrombotic events associated with the concomitant use of emicizumab and APCC remains to be clarified, however.

The present study was designed, therefore, to investigate the role of APCR or ATR in VTEs induced by the concomitant use of emicizumab and APCCs in persons with hemophilia A. Thrombin generation assays (TGAs) were used to assess APCR and ATR in pooled normal plasma (PNP) and in FVIII-deficient (FVIIIdef) plasma in the presence of emicizumab and APCCs.

## 2 Methods

Blood samples from healthy volunteers were collected in accordance with the ethical guidelines of Nara Medical University, and the collection of normal individual samples was approved by the Medical Research Ethics Committee (Approval No. 2505). Their blood samples were obtained after informed consent following local ethical guidelines.

### 2.1 Reagents

Emicizumab (Hemlibra) [17,18], rFVIIa (NovoSeven), APCC (FEIBA), and plasma-derived (pd)FVIIa/FX (Byclot; *see below*) were obtained from Chugai Pharmaceutical Co, Novo Nordisk A/S, Takeda Pharmaceutical Co, Ltd, and KM Biologics Co, Ltd, respectively. Recombinant human tissue factor (Innovin, Dade), FVIIIdef plasma (George King Biomedical Inc), pd-APC, pd-AT, pd-FII, pd-FIXa (Haematologic Technologies Inc), a thrombin-specific fluorogenic substrate (Z-Gly-Gly-Arg-AMC, Bachem) were purchased from the indicated vendors. Phospholipid (PL) vesicles containing 10% phosphatidylserine, 60% phosphatidylcholine, and 30% phosphatidylethanolamine were prepared as previously described [29].

### 2.2 Plasma samples

PNP was prepared from 20 normal healthy individuals. Whole blood samples were collected in plastic tubes containing 3.2% sodium citrate at a ratio of 9:1 (Fuso Pharmaceutical Industries). No study subjects had taken any medication that may have influenced platelet or coagulation function 2 weeks prior to blood sampling. Platelet-poor plasma was separated by centrifuging citrated whole blood for 10 minutes at 2000 × *g*. All plasma samples were stored at −80 °C and thawed at 37 °C immediately prior to the assays.

### 2.3 Preparation of plasma samples containing emicizumab and BPAs

Recommended guidelines for on-demand BPA therapy during emicizumab prophylaxis have been published [26]. In addition, pd-FVIIa/FX, consisting of a mixture of FVIIa and FX at a weight ratio of 1:10, has been approved in Japan since 2014 for persons with congenital hemophilia with inhibitors and acquired hemophilia [30,31]. Previous studies have also reported that pd-FVIIa/FX administration at a dose of 60 μg/kg could increase FVIIa and FX levels in plasma to 14 nM and 293 nM, respectively [32,33]. BPAs such as APCC at 1.3 IU/mL (corresponding to 50 IU/kg), rFVIIa at 2.2 μg/mL (corresponding to 90 μg/kg), and pd-FVIIa/FX at 1.5 μg/mL (corresponding to 60 μg/kg) were added to the FVIIIdef plasmas in the presence of emicizumab (50 μg/mL).

### 2.4 TGAs

TGAs were performed as previously described [34]. Briefly, plasma samples (80 μL) were preincubated for 10 minutes with 20 μL of a trigger reagent containing tissue factor and PL (final concentrations, 0.5 pM and 4 μM, respectively). After adding 20 μL of a reagent containing CaCl_2_ and fluorogenic substrate (final concentrations, 16.7 mM and 2.5 mM, respectively), the development of fluorescent signals was monitored using a Fluoroskan Ascent microplate reader (Thermo Fisher Scientific). Data analyses were performed using the manufacturer’s software to derive the standard parameters: peak thrombin (PeakTh) and endogenous thrombin potential (ETP). The PeakTh and ETP values obtained from 15 healthy individuals were 312 ± 76 nM and 2805 ± 231 nM × min, respectively. The PeakTh inhibition was calculated as follows: ([PeakTh in PNP or FVIIIdef plasma spiked with emicizumab and BPAs without APC − PeakTh in PNP or FVIIIdef plasma spiked with emicizumab and BPAs with APC at 8 or 16 nM]/[PeakTh in PNP or FVIIIdef plasma spiked with emicizumab and BPAs without APC]) × 100. The percentage inhibition of the PeakTh value in PNP was approximately 30% when APC 8 nM was added or that was approximately 40% when APC 16 nM was added. These percentage inhibition values were considered normal values. Based on these results, if the percentage inhibition of the PeakTh value was <15% when APC 8 nM was added or that was <20% when APC 16 nM was added, we defined it as APCR.

### 2.5 Statistical analysis

Experiments were performed 3 times, and data were presented as the average and SD. Data analysis was performed using KaleidaGraph (Synergy). Significant differences were determined by Dunnett’s multiple comparison test. *P* values <.05 were considered statistically significant.

## 3 Results

### 3.1 Assessment of AT-mediated downregulation in FVIIIdef plasma mixed with emicizumab and BPAs

**Supplemental Figure 1:**
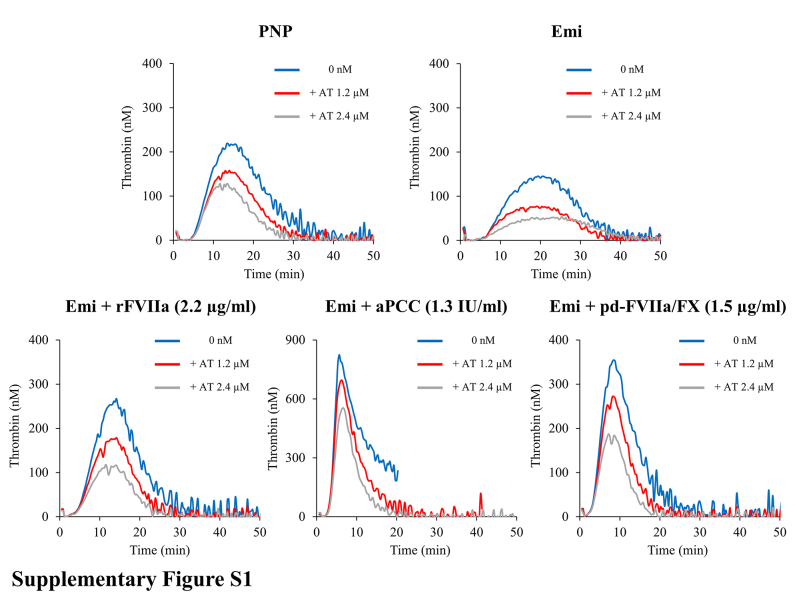
**Impact of emicizumab and FVIII-bypassing agents on AT-induced down regulation in pooled normal plasma (PNP) or FVIII-deficient plasma.**TF-triggered thrombin generation was assessed after the addition of various concentrations of AT (0-2.4 µM) to PNP or FVIII-deficient plasma supplemented with emicizumab (50 µg/mL) alone, emicizumab and bypassing agents (rFVIIa 2.2 µg/mL, aPCC 1.3 IU/mL, pd-FVIIa/FX 1.5 µg/mL). Experiments were performed three times and the representative AT dose-dependent thrombin generation curves are shown (*blue*; no AT, *red*; 1.2 µM AT, *gray*; 2.4 µM AT).

First, TGAs were utilized to evaluate AT-mediated downregulation after the addition of AT to PNP and FVIIIdef plasmas spiked with emicizumab and BPAs. Representative curves are shown in Supplementary Figure S1. The addition of AT to PNP and FVIIIdef plasma supplemented with emicizumab alone significantly decreased PeakTh and ETP values. Also, the addition of AT (1.2 and 2.4 μM) to FVIIIdef plasma spiked with emicizumab and rFVIIa (2.2 μg/mL) or pd-FVIIa/FX (1.5 μg/mL) significantly lowered ETP, and the addition of AT at 2.4 μM significantly decreased their PeakTh compared with those without AT (Table 1). In the presence of emicizumab and APCC (1.3 IU/mL), the PeakTh in FVIIIdef plasma exceeded that in the copresence of emicizumab and rFVIIa or pd-FVIIa/FX as previously demonstrated [35]. Furthermore, the addition of AT (1.2 and 2.4 μM) decreased the PeakTh in FVIIIdef plasmas spiked with emicizumab and APCC. ETP could not be determined in plasma containing emicizumab and APCC in the absence of exogenous AT (Table 1). These findings indicated that ATR was not associated with mixtures of emicizumab and APCC.

**Table 1:** Parameters in pooled normal plasma or factor (F)VIII–deficient plasmas supplemented with emicizumab and FVIII-bypassing agents in the presence of antithrombin.

	PeakTh	ETP		PeakTh	ETP		PeakTh	ETP		PeakTh	ETP		PeakTh	ETP
	nM	nM × min		nM	nM × min		nM	nM × min		nM	nM × min		nM	nM × min
PNP	251 ± 45	3505 ± 91	Emi	160 ± 22	3022 ± 155	Emi + rFVIIa	308 ± 12	3924 ± 133	Emi + pd-FVIIa/FX	401 ± 8	4258 ± 176	Emi + APCC	791 ± 31	N.D.
PNP (1.2)	137 ± 26	2230 ± 17^a^	Emi (1.2)	78 ± 4^b^	1794 ± 348^b^	Emi + rFVIIa (1.2)	204 ± 38	2352 ± 291^b^	Emi + pd-FVIIa/FX (1.2)	298 ± 38	2555 ± 357^b^	Emi + APCC (1.2)	689 ± 7^b^	4815 ± 334
PNP (2.4)	102 ± 29^b^	1667 ± 34^a^	Emi (2.4)^a^	52 ± 2^a^	1371 ± 116^a^	Emi + rFVIIa (2.4)	144 ± 47^b^	1709 ± 444^a^	Emi + pd-FVIIa/FX (2.4)	218 ± 53^b^	1807 ± 464^b^	Emi + APCC (2.4)	573 ± 27^a^	3604 ± 186

Tissue factor–triggered thrombin generation assays were performed as described in Methods. The parameters obtained after the addition of antithrombin (AT) to PNP or FVIII-deficient plasmas spiked with Emi (50 μg/mL) alone, Emi and FVIII-bypassing agent (rFVIIa at 2.2 μg/mL, APCC at 1.3 IU/mL, and pd-FVIIa/FX at 1.5 μg/mL) are shown. Thrombin generation assay parameters in the presence of AT were compared with those in the absence of AT. Significant differences were considered as *P* < .05. One sample in each subgroup was tested per experiment. Experiments were performed 3 times, and the average values and SD are shown. The PeakTh and ETP values obtained from 15 healthy individuals were 312 ± 76 nM and 2805 ± 231 nM × min, respectively, as described in Methods. The figures in parentheses 1.2 and 2.4 indicate +AT 1.2 μM and +AT 2.4 μM, respectively.

APCC, activated prothrombin complex concentrate; Emi, emicizumab; ETP, endogenous thrombin potential; F, factor; pd, plasma-derived; N.D., not detected; PeakTh, peak thrombin; PNP, pooled normal plasma; rFVIIa, recombinant activated factor VII.

a *P* < .01 vs no AT.

b *P* < .05 vs no AT.

### 3.2 Assessment of APCR in FVIIIdef plasma mixed with emicizumab and APCC

**Supplemental Figure 2:**
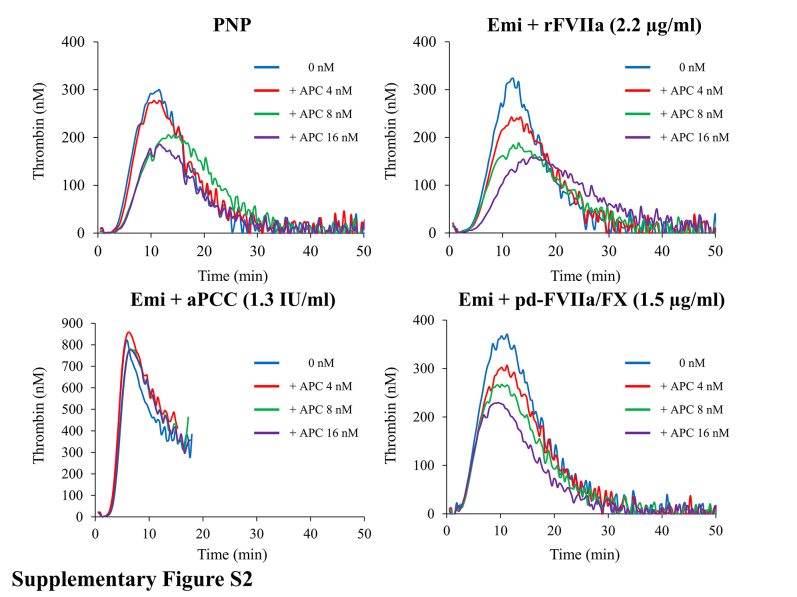
**Effects of APC in pooled normal plasma (PNP) or FVIII-deficient plasma spiked with emicizumab and FVIII-bypassing agents.** TF-triggered thrombin generation in the presence of various concentrations of APC (0–16 nM) was examined in PNP and FVIII-deficient plasmas supplemented with emicizumab (50 µg/mL) and bypassing agents (rFVIIa 2.2 µg/mL, aPCC 1.3 IU/mL, pd-FVIIa/FX 1.5 µg/mL). Experiments were performed three times and representative APC dose-dependent thrombin generation curves are shown (*blue*; no APC, *red*; APC 4 nM, *green*; APC 8 nM, *purple*; APC 16 nM).

**Supplemental Figure 3:**
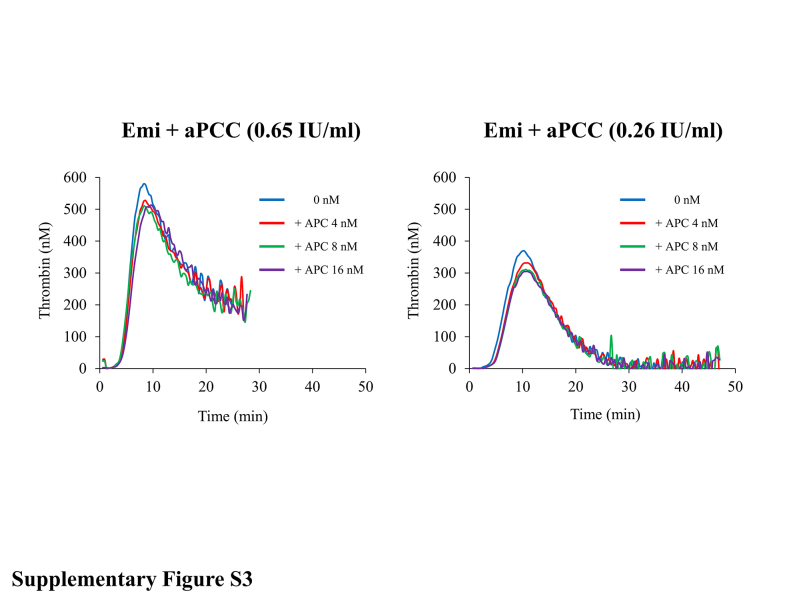
**APC-induced downregulation of thrombin generation in FVIII-deficient plasma spiked with emicizumab and low dose of aPCC**. TF-triggered thrombin generation after the addition of various concentrations of APC (0–16 nM) was examined in FVIII-deficient plasmas supplemented with emicizumab (50 µg/mL) and aPCC (0.26 or 0.65 IU/mL). Experiments were performed three times and representative APC dose-dependent thrombin generation curves are shown (*blue*; no APC, *red*; APC 4 nM, *green*; APC 8 nM, *purple*; APC 16 nM).

**Supplemental Figure 4:**
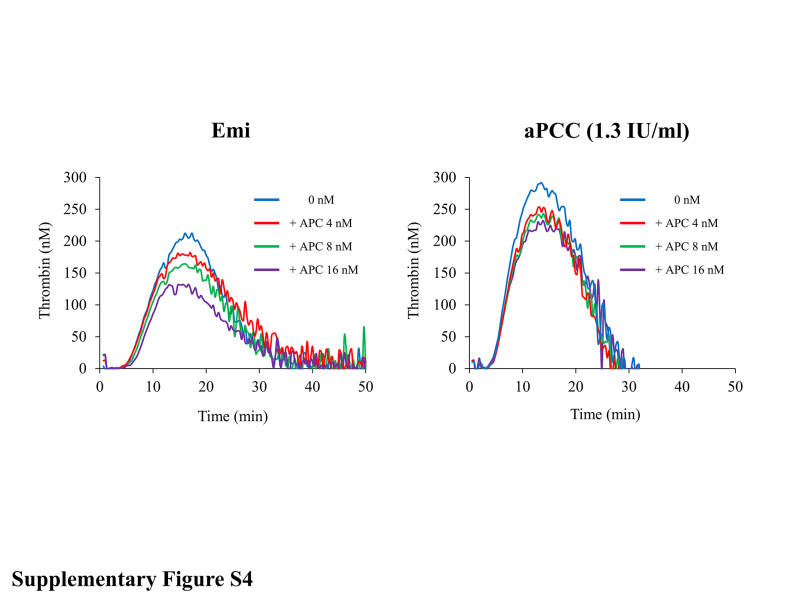
**APC-induced downregulation of thrombin generation in FVIII-deficient plasma spiked with emicizumab or aPCC.** TF-triggered thrombin generation was assessed after the addition of various concentrations of APC (0–16 nM) to FVIII-deficient plasmas supplemented with emicizumab (50 µg/mL) or aPCC (1.3 IU/mL). Experiments were performed three times and representative APC dose-dependent thrombin generation curves are shown (*blue*; no APC, *red*; APC 4 nM, *green*; APC 8 nM, *purple*; APC 16 nM).

We next examined APC-mediated inactivation of hemostasis in FVIIIdef plasma incubated with emicizumab and each BPA. The addition of APC (8 and 16 nM) to PNP and to FVIIIdef plasma samples spiked with emicizumab and rFVIIa or pd-FVIIa/FX significantly reduced their PeakTh value (Supplementary Figure S2). In each instance, PeakTh was reduced by approximately 30% at 8 nM APC and 40% at 16 nM APC, respectively (Table 2). The measurements of ETP were also significantly lower at 16 nM APC. In contrast, APC did not reduce PeakTh effectively at any concentration in the copresence of emicizumab and APCC (Supplementary Figure S2), indicating the APCR in the copresence of emicizumab and APCC. APC also did not significantly decrease PeakTh in FVIIIdef plasmas mixed with emicizumab and APCC at 0.65 or 0.26 IU/mL (Supplementary Figure S3 and Table 3). To clarify whether APCR in both emicizumab and APCC attributed to either emicizumab or APCC, we investigated the APC-mediated inactivation in FVIIIdef plasmas spiked with emicizumab (50 μg/mL) or APCC (1.3 IU/mL) alone (Supplementary Figure S4). In the presence of emicizumab alone, APC (16 nM) significantly reduced PeakTh as previously described [36]. The percentage inhibition of PeakTh by APC at 16 nM in emicizumab alone was 41%, similar to the PNP. In the presence of APCC alone, however, PeakTh and ETP of thrombin generation were not significantly depressed by APC. PeakTh was reduced by 15% at 8 nM and 19% at 16 nM APC, respectively (Supplementary Table S1 and Table 4). On the other hand, the percentage inhibition achieved with APC at 8 or 16 nM in emicizumab and APCC (1.3 IU/mL) was 0% or 5%, respectively (Table 4). In addition, the APCR in emicizumab and APCC (1.3 IU/mL) was statistically different from that in APCC (1.3 IU/mL) alone (data not shown). Taken together, these results suggest that APCC (1.3 IU/mL) alone exhibited the partial APCR and that the APCR in copresence of emicizumab and APCC was enhanced relative to that in the presence of APCC alone.

**Table 2:** Activated protein C–mediated inactivation in pooled normal plasma or in factor (F)VIII–deficient plasma supplemented with emicizumab and FVIII-bypassing agents.

	PeakTh	ETP		PeakTh	ETP		PeakTh	ETP		PeakTh	ETP
	nM	nM × min		nM	nM × min		nM	nM × min		nM	nM × min
PNP	291 ± 8	3307 ± 337	Emi + rFVIIa	290 ± 44	3717 ± 14	Emi + pd-FVIIa/FX	355 ± 11	4588 ± 419	Emi + APCC	774 ± 48	N.D.
PNP (4)	254 ± 34	3471 ± 180	Emi + rFVIIa (4)	233 ± 12	3474 ± 99	Emi + pd-FVIIa/FX (4)	311 ± 14^a^	4050 ± 307	Emi + APCC (4)	807 ± 70	N.D.
PNP (8)	190 ± 19^a^	3146 ± 292	Emi + rFVIIa (8)	179 ± 4^a^	3500 ± 30	Emi + pd-FVIIa/FX (8)	264 ± 4^b^	3619 ± 218	Emi + APCC (8)	776 ± 7	N.D.
PNP (16)	170 ± 15^a^	2384 ± 47^a^	Emi + rFVIIa (16)	168 ± 17^a^	3174 ± 105^b^	Emi + pd-FVIIa/FX (16)	221 ± 13^b^	2970 ± 123^a^	Emi + APCC (16)	736 ± 55	N.D.

Tissue factor–triggered thrombin generation assays were performed as described in Methods. The parameters obtained after the addition of activated protein C (APC) to PNP or FVIII-deficient plasmas spiked with Emi (50 μg/mL) and FVIII-bypassing agent (rFVIIa, 2.2 μg/mL; APCC, 1.3 IU/mL; pd-FVIIa/FX, 1.5 μg/mL) are shown. TGA parameters in the presence of APC were compared with those in the absence of APC. Significant differences were considered as *P* < .05. One sample in each subgroup was tested per experiment. Experiments were performed 3 times, and the average values and SD are shown. The PeakTh and ETP values obtained from 15 healthy individuals were 312 ± 76 nM and 2805 ± 231 nM × min, respectively, as described in Methods. The figures in parentheses 4, 8, and 16 indicate +APC 4 nM, +APC 8 nM, and +APC 16 nM, respectively.

APCC, activated prothrombin complex concentrate; Emi, emicizumab; ETP, endogenous thrombin potential; F, factor; N.D., not detected; pd, plasma-derived; PeakTh, peak thrombin; PNP, pooled normal plasma; rFVIIa, recombinant activated factor VII.

a *P* < .05 vs no APC.

b *P* < .01 vs no APC.

**Table 3:** Activated protein C–mediated inactivation in factor VIII–deficient plasma supplemented with emicizumab and low dose of activated prothrombin complex concentrate.

	PeakTh	ETP		PeakTh	ETP
	nM	nM × min		nM	nM × min
Emi + APCC 0.65 IU/mL	576 ± 4	N.D.	Emi + APCC 0.26 IU/mL	379 ± 15	3879 ± 128
Emi + APCC 0.65 IU/mL (4)	556 ± 44	N.D.	Emi + APCC 0.26 IU/mL (4)	348 ± 22	3737 ± 130
Emi + APCC 0.65 IU/mL (8)	533 ± 41	N.D.	Emi + APCC 0.26 IU/mL (8)	335 ± 33	3722 ± 506
Emi + APCC 0.65 IU/mL (16)	517 ± 6	N.D.	Emi + APCC 0.26 IU/mL (16)	319 ± 18	3688 ± 443

Tissue factor–triggered thrombin generation assays were performed as described in Methods. The parameters obtained after the addition of activated protein C (APC) to factor VIII–deficient plasmas spiked with Emi (50 μg/mL) and APCC (0.26 or 0.65 IU/mL) are shown. TGA parameters in the presence of APC were compared with those in the absence of APC. Significant differences were considered as *P* < .05. One sample in each subgroup was tested per experiment. Experiments were performed 3 times, and the average values and SD are shown. The PeakTh and ETP values obtained from 15 healthy individuals were 312 ± 76 nM and 2,805 ± 231 nM × min, respectively, as described in Methods. The figures in parentheses 4, 8, and 16 indicate +APC 4 nM, +APC 8 nM, and +APC 16 nM, respectively.

APCC, activated prothrombin complex concentrate; Emi, emicizumab; ETP, endogenous thrombin potential; N.D., not detected; PeakTh, peak thrombin.

**Table 4:** The percentage inhibition of peak thrombin achieved with activated protein C in pooled normal plasma or in factor (F)VIII–deficient plasma supplemented with emicizumab and FVIII-bypassing agents.

	PNP		Emi		APCC (1.3)		Emi + rFVIIa		Emi + pd-FVIIa/FX		Emi + APCC (1.3)		Emi + APCC (0.65)		Emi + APCC (0.13)
	%		%		%		%		%		%		%		%
APC (8)	35 ± 3	APC (8)	26 ± 12	APC (8)	15 ± 2	APC (8)	38 ± 6	APC (8)	26 ± 2	APC (8)	0 ± 0.3	APC (8)	9 ± 5	APC (8)	12 ± 4
APC (16)	42 ± 2	APC (16)	41 ± 9	APC (16)	19 ± 3	APC (16)	41 ± 10	APC (16)	38 ± 2	APC (16)	5 ± 1	APC (16)	12 ± 2	APC (16)	16 ± 2

Tissue factor–triggered thrombin generation assays were performed as described in Methods. The percentage inhibition of peak thrombin obtained after the addition of APC to PNP or FVIII-deficient plasmas spiked with Emi (50 μg/mL) and FVIII-bypassing agent (rFVIIa, 2.2 μg/mL; APCC, 0.13 or 0.65 or 1.3 IU/mL; pd-FVIIa/FX, 1.5 μg/mL) is shown. The figures in parentheses APC (8) and APC (16) indicate +APC 8 nM and +APC 16 nM, respectively. The figures in parentheses APCC (0.13), APCC (0.65), and APCC (1.3) indicate APCC 0.13 IU/mL, APCC 0.65 IU/mL, and APCC 1.3 IU/mL, respectively.

APC, activated protein C; APCC, activated prothrombin complex concentrate; Emi, emicizumab; F, factor; pd, plasma-derived; PNP, pooled normal plasma; rFVIIa, recombinant activated factor VII.

### 3.3 APCR in FVIIIdef plasma mixed with emicizumab and FIXa or FII

**Supplemental Figure 5:**
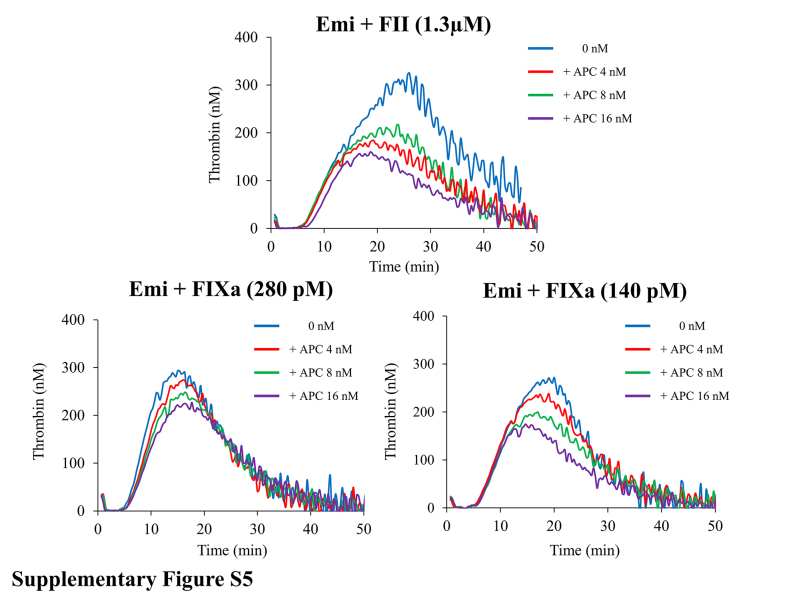
**APC-induced downregulation of thrombin generation in FVIII-deficient plasma spiked with emicizumab and FII or FIXa.** TF-triggered thrombin generation after the addition of various concentrations of APC (0–16 nM) was examined in FVIII-deficient plasmas supplemented with emicizumab (50 µg/mL) and FII (1.3 µM) or FIXa (140 or 280 pM). Experiments were performed three times and representative APC dose-dependent thrombin generation curves are shown (*blue*; no APC, *red*; APC 4 nM, *green*; APC 8 nM, *purple*; APC 16 nM).

Previous studies have demonstrated that PeakTh generation, measured by TGA in the presence of emicizumab and APCC, could be associated with the concentrations of FIXa and prothrombin (FII) in APCC [37]. APC-mediated downregulation in FVIIIdef plasma was investigated, therefore, in FVIIIdef plasma samples supplemented with emicizumab and FIXa or FII. Plasma concentrations of FIXa and FII were based on the earlier evidence that APCC (1 IU/mL) contained 280 pM FIXa and 1.3 μM FII [37]. The addition of APC (4-16 nM) to plasma incubated with emicizumab and FII significantly reduced the PeakTh to similar levels as in PNP (Supplementary Figure S5), with 45% reduction at 16 nM APC (Tables 5 and 6). In contrast, both PeakTh and ETP were not significantly affected in the presence of emicizumab with 280 pM FIXa (Supplementary Figure S5). PeakTh levels were reduced by <20% in the presence of 16 nM APC in these plasma samples, indicating that APC-mediated reactions were weaker compared with PNP (Tables 5 and 6). However, further experiments using emicizumab together with a lower concentration of FIXa (140 pM) demonstrated that the addition of APC (16 nM) significantly reduced both PeakTh and ETP (Supplementary Figure S5; Tables 5 and 6), suggesting that APC-catalyzed inactivation was observed under these conditions. The results supported that the presence of FIXa in APCC contributed to the APCR in the concomitant use of emicizumab and APCC.

**Table 5:** Activated protein C–mediated inactivation in factor (F)VIII–deficient plasma supplemented with emicizumab and FII or FIXa.

	PeakTh	ETP		PeakTh	ETP		PeakTh	ETP
	nM	nM × min		nM	nM × min		nM	nM × min
Emi + FII	309 ± 4	N.D.	Emi + FIXa 280 pM	281 ± 10	3895 ± 1114	Emi + FIXa 140 pM	260 ± 8	4648 ± 155
Emi + FII (4)	225 ± 29^a^	N.D.	Emi + FIXa 280 pM (4)	274 ± 4	4011 ± 787	Emi + FIXa 140 pM (4)	211 ± 30	4269 ± 524
Emi + FII (8)	180 ± 3^b^	3501 ± 1143	Emi + FIXa 280 pM (8)	260 ± 23	3931 ± 614	Emi + FIXa 140 pM (8)	182 ± 18^a^	3743 ± 317
Emi + FII (16)	169 ± 18^b^	3008 ± 651	Emi + FIXa 280 pM (16)	233 ± 16	4004 ± 627	Emi + FIXa 140 pM (16)	159 ± 17^a^	2949 ± 280^a^

Tissue factor–triggered thrombin generation assays were performed as described in Methods. The parameters obtained after the addition of activated protein C (APC) to FVIII-deficient plasmas spiked with Emi (50 μg/mL) and FII (1.3 μM) or FIXa (140 or 280 pM) are shown. Thrombin generation assay parameters in the presence of APC were compared with those in the absence of APC. Significant differences were considered as *P* < .05. One sample in each subgroup was tested per experiment. Experiments were performed 3 times, and the average values and SD are shown. The PeakTh and ETP values obtained from 15 healthy individuals were 312 ± 76 nM and 2,805 ± 231 nM × min, respectively, as described in Methods. The figures in parentheses 4, 8, 16 indicate +APC 4 nM, +APC 8 nM, and +APC 16 nM, respectively.

Emi, emicizumab; ETP, endogenous thrombin potential; F, factor; N.D., not detected; PeakTh, peak thrombin.

a *P* < .05 vs no APC.

b *P* < .01 vs no APC.

**Table 6:** The percentage inhibition of peak thrombin achieved with activated protein C in pooled normal plasma or factor (F)VIII–deficient plasma supplemented with emicizumab and FII or FIXa and supplemented with emicizumab and FIXa and FII or FVIII-bypassing agents.

	Emi + FII		Emi + FIXa (140)		Emi + FIXa (280)		Emi + FIXa (280) + rFVIIa		Emi + FIXa (280) + pd-FVIIa/FX		Emi + FIXa (280) + FII
	%		%		%		%		%		%
APC (8)	42 ± 2	APC (8)	30 ± 4	APC (8)	7 ± 1	APC (8)	16 ± 3	APC (8)	15 ± 3	APC (8)	2 ± 1
APC (16)	45 ± 4	APC (16)	39 ± 4	APC (16)	17 ± 6	APC (16)	26 ± 2	APC (16)	19 ± 2	APC (16)	8 ± 2

Tissue factor–triggered thrombin generation assays were performed as described in Methods. The percentage inhibition of peak thrombin obtained after the addition of APC to FVIII-deficient plasmas spiked with Emi (50 μg/mL) and FII (1.3 μM) or FIXa (140 or 280 pM) and spiked with Emi (50 μg/mL) and FIXa (280 pM) and FVIII-bypassing agent (rFVIIa 2.2 μg/mL, pd-FVIIa/FX 1.5 μg/mL) or FII (1.3 μM) is shown. The figures in parentheses APC (8) and APC (16) indicate +APC 8 nM and +APC 16 nM, respectively. The figures in parentheses FIXa (140) and FIXa (280) indicate FIXa 140 pM and FIXa 280 pM, respectively.

APC, activated protein C; Emi, emicizumab; F, factor; pd, plasma-derived; rFVIIa, recombinant activated factor VII.

### 3.3 APCR in FVIIIdef plasma mixed with emicizumab together with separate constituents of APCC

**Supplemental Figure 6:**
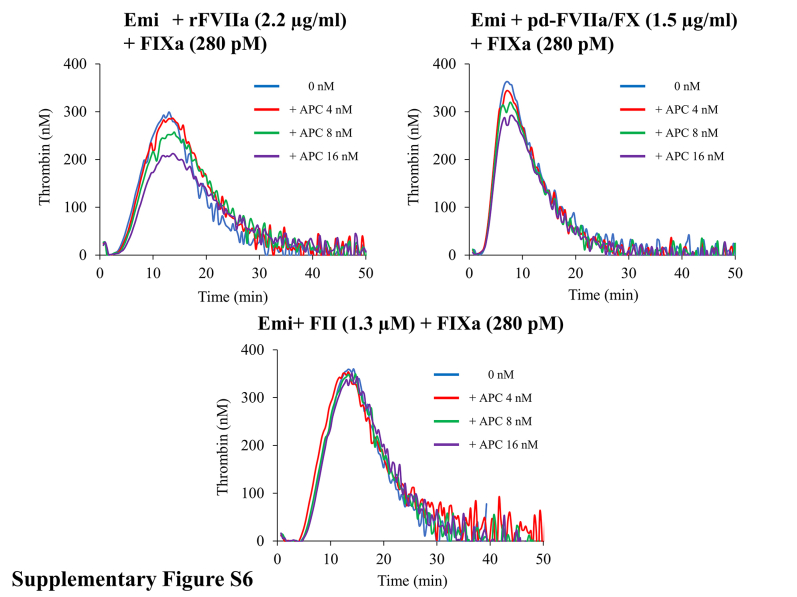
**APC-mediated downregulation of thrombin generation in FVIII-deficient plasma spiked with emicizumab, FIXa, and rFVIIa or pd-FVIIa/FX or FII.** TF-triggered thrombin generation after the addition of various concentrations of APC (0-16 nM) was examined in FVIII-deficient plasmas supplemented with emicizumab (50 µg/mL), FIXa (280 pM), and rFVIIa (2.2 µg/mL) or pd-FVIIa/FX (1.5 µg/mL) or FII (1.3 µM). Experiments were performed three times and representative APC dose-dependent thrombin generation curves are shown (*blue*; no APC, *red*; APC 4 nM, *green*; APC 8 nM, *purple*; APC 16 nM).

As noted above, FIXa in APCC seemed to be a key component linked to APCR in the combination of emicizumab with APCC, and APC-mediated inactivation in mixtures of emicizumab and rFVIIa or pd-FVIIa/FX in the absence of FIXa was close to the PNP (Tables 2 and 4). Investigations were continued, therefore, to examine whether the addition of FIXa could influence APC-catalyzed inactivation in the copresence of emicizumab and rFVIIa or pd-FVIIa/FX. The results indicated that APC (4-16 nM) did not significantly reduce PeakTh and ETP in FVIIIdef plasmas containing emicizumab and rFVIIa or pd-FVIIa/FX together with FIXa (Supplementary Figure S6), although levels of PeakTh were moderately reduced (20%-25%) after the addition of APC (16 nM) (Tables 6 and 7). In addition, we evaluated the APC-catalyzed inactivation in the copresence of emicizumab, FIXa, and FII. Although the PeakTh in the copresence of emicizumab, FIXa, and FII was lower than that in the copresence emicizumab and APCC, TGA results showed that the copresence of emicizumab, FIXa, and FII was almost resistant to APC-mediated inactivation, as well as the copresence of emicizumab and APCC (Supplementary Figure S6). The percentage inhibition of PeakTh by APC (16 nM) in the copresence of emicizumab, FIXa, and FII was 8%, indicating an effect almost similar to that in the copresence of emicizumab and APCC at 1.3 IU/mL (Tables 4 and 6). Taken together, our findings suggested that both FIXa and FII were principal clotting factors associated with APCR in the combination of emicizumab with APCC.

**Table 7:** Parameters in factor (F)VIII–deficient plasmas supplemented with emicizumab, FIXa, and recombinant activated FVII or plasma-derived FVIIa/FX or FII in the presence of activated protein C.

	PeakTh	ETP		PeakTh	ETP		PeakTh	ETP
	nM	nM × min		nM	nM × min		nM	nM × min
Emi + FIXa + rFVIIa	278 ± 21	3432 ± 628	Emi + FIXa + pd-FVIIa/FX	419 ± 77	4159 ± 606	Emi + FII + FIXa	347 ± 17	4956 ± 812
Emi + FIXa + rFVIIa (4)	253 ± 45	3525 ± 1112	Emi + FIXa + pd-FVIIa/FX (4)	378 ± 47	4039 ± 817	Emi + FII + FIXa (4)	339 ± 15	5435 ± 126
Emi + FIXa + rFVIIa (8)	234 ± 29	3676 ± 747	Emi + FIXa + pd-FVIIa/FX (8)	354 ± 50	3856 ± 813	Emi + FII + FIXa (8)	340 ± 8	4944 ± 471
Emi + FIXa + rFVIIa (16)	206 ± 12	3500 ± 303	Emi + FIXa + pd-FVIIa/FX (16)	341 ± 72	4001 ± 1303	Emi + FII + FIXa (16)	319 ± 27	4941 ± 474

Tissue factor–triggered thrombin generation assays were performed as described in Methods. The parameters obtained after the addition of activated protein C (APC) to FVIII-deficient plasmas spiked with Emi (50 μg/mL), FIXa (280 pM), and rFVIIa (2.2 μg/mL) or pd-FVIIa/FX (1.5 μg/mL) or FII (1.3 μM) are shown. Thrombin generation assay parameters in the presence of APC were compared with those in the absence of APC. Significant differences were considered as *P* < .05. One sample in each subgroup was tested per experiment. Experiments were performed 3 times, and the average values and SD are shown. The PeakTh and ETP values obtained from 15 healthy individuals were 312 ± 76 nM and 2805 ± 231 nM × min, respectively, as described in Methods. The figures in parentheses 4, 8, and 16 indicate +APC 4 nM, +APC 8 nM, and +APC 16 nM, respectively.

Emi, emicizumab; ETP, endogenous thrombin potential; F, factor; pd, plasma-derived; PeakTh, peak thrombin; rFVIIa, recombinant activated factor VII.

## 4 Discussion

Persons with hemophilia A with FVIII inhibitors being treated with emicizumab commonly require supplementary BPAs, including rFVIIa and APCC, for breakthrough bleeding or surgical management. Thrombotic microangiopathy (TMA) and VTEs have been reported after the concomitant use of emicizumab and APCC [21], however, and consequently, the use of rFVIIa has been recommended as first-line treatment in these patients. Clinical reports have suggested that the increased thrombotic risk might be associated with enhanced thrombin generation potentiated by FIX and FX and their activated forms present in APCC [6]. Alternatively, emicizumab is not inactivated by natural anticoagulants that control hemostasis, including AT, APC, and other regulatory systems [38], and the precise mechanism(s) that causes VTEs remains to be clarified. The present study demonstrated that emicizumab and APCC concomitant therapy increased APCR due to the presence of FIXa and FII in APCC.

Our data showed that emicizumab alone did not interfere with anticoagulant function of AT as previously described [39]. However, the effect of AT in FVIIIdef plasmas spiked with emicizumab and BPAs such as rFVIIa, APCC, or pd-FVIIa/FX has not been investigated. Our initial experiments confirmed that AT-mediated inactivation of coagulation was not restricted in the copresence of emicizumab and any BPA. Thrombotic complications were not prevalent in emicizumab-treated persons with hemophilia A receiving single or infrequent doses of APCCs, supporting that AT-mediated downregulation would prevent the development of VTEs in persons with hemophilia A treated with emicizumab and infrequent doses of APCCs. Our investigations provided further information, however, on the important relationships between APCR and VTEs. The APC-mediated downregulation in the copresence of emicizumab and rFVIIa or pd-FVIIa/FX was comparable with that in PNP, although the inactivation by APC was ineffective to FVIIIdef plasmas in the presence of emicizumab and APCC. The pd-FVIIa/FX (1.5 μg/mL) used in these experiments contains much higher concentrations of FVIIa and FX than those in normal plasma, and it seemed, therefore, that FVIIa and FX would not be associated with the APCR observed with APCC.

Moreover, our results suggested that the addition of FIXa at 280 pM and emicizumab in FVIIIdef plasmas could lead to a partial APCR state, but was not affected at 140 pM FIXa. In addition, the APCR in the copresence of emicizumab and rFVIIa or pd-FVIIa/FX was not also shown. These results indicated that APCR is observed in the presence of both emicizumab and over a certain level of FIXa. We speculate that the reduced potency of APC in the presence of emicizumab and FIXa at 280 pM could be due to increased FXa generated from FIXa-emicizumab-FX complexes. Hence, this FXa could result in enhanced FXa/FVa/FVII (prothrombinase complex) formation, and accelerated conversion of FII to thrombin [40]. Also, an earlier report indicated that emicizumab enhanced the binding of FX to FIXa but had a limited allosteric improvement of the FIXa active site [41]. Therefore, the combination of FIXa at 140 pM with emicizumab may not result in APCR. APCR was not observed with FVIIIdef plasma in the presence of emicizumab and FII (1.3 μM). Notably, however, similar plasma samples mixed with emicizumab and both FIXa (280 pM), and FII (1.3 μM) exhibited more APCR potential than those mixed with emicizumab and FIXa alone. The mechanism(s) by which emicizumab, FIXa (280 pM), and FII (1.3 μM) were near-completely resistant to the APC reaction may be that the prothrombinase complex increases by generating more FXa from FIXa-emicizumab-FX and that those prothrombinase complexes and additional FII could produce the large amounts of thrombin because of their synergistic effect. Earlier investigations on the clinical use of APCC-related products suggested that a complex of FII and FXa induced a synergistic effect on thrombin generation relative to FII or FXa alone [42]. These data support our hypothesis that additional FII potentiates the coactive generation of FXa in the prothrombinase complex comprising FIXa-emicizumab-FX, leading to enhanced thrombin production. The APCR potential in the copresence of emicizumab, FIXa (280 pM), and rFVIIa or pd-FVIIa/FX was weaker than that in emicizumab, FIXa (280 pM) and FII (1.3 μM), consistent with our hypothesis. Consequently, the consensus appears to support the concept that FIXa and FII are key clotting factors for APCR in persons with hemophilia A with inhibotors treated with emicizumab and APCC.

As discussed above, analysis of the HAVEN 1 phase 3 clinical trial data highlighted that TMA were associated with the clinical use of emicizumab in combination with APCC [21]. More recently, Valls et al. [43] confirmed that a combination of APCC or rFVIIa with emicizumab is related to the activation of the coagulation cascade and that disturbances in other functional processes, including the complement system, fibrinogenesis (fibrinogen α, β, and γ chain), and the major platelet adhesive receptor glycoproteins (GP1bα, GPV, and GPIX), might explain the occurrence of drug-induced TMA in some persons with hemophilia A treated with emicizumab plus APCC. In terms of coagulation factors, FIX/FIXa appeared to be involved with the mechanism(s) linking APCC to the occurrence of TMA [43]. Moreover, the previous study reported that FIXa in APCC could result in excessive thrombin generation in the copresence of emicizumab and APCC [37]. Our data also demonstrated that FIXa is a key component in APCR of emicizumab and APCC. Taken together, FIXa in APCC could be the most important coagulation factor for the development of both TMA and VTEs in persons with hemophilia A receiving emicizumab and APCC.

The findings of the present study are limited by the failure to identify why the incubation of FVIIIdef plasma with APCC alone exhibited APCR to some extent. It may be that diverse physiological interactions recently explained in the previous report [43] contribute to the clinical pathology, and further studies are warranted in this respect. In addition, effects of APC and AT on thrombin generation appeared to be minor and the relative change in ETP or PeakTh may be dependent on the BPAs because the combination of emicizumab and different BPAs exhibit different ETP and PeakTh, and the PeakTh in the combination of emicizumab with APCC was especially high. Nevertheless, we demonstrated that FIXa and FII could contribute to APCR and the enhanced risk for VTEs in emicizumab-treated persons with hemophilia A with inhibitors. We hope that our data could be helpful in preventing VTEs in emicizumab-treated persons with hemophilia A with inhibitors.
