# Supplementary material for: Activated protein C resistance in the copresence of emicizumab and activated prothrombin complex concentrates
Source: Res Pract Thromb Haemost. 2024 Jun 18;8(4):102479. doi: 10.1016/j.rpth.2024.102479 (PMC11305313; doi:10.1016/j.rpth.2024.102479)
Supplement: Supplemental Table S1 [file mmc1.docx]

**Supplementary Table S1. APC-mediated inactivation in FVIII-deficient plasma supplemented with emicizumab or aPCC**

TF-triggered thrombin generation assays (TGA) were performed as described in Methods. The parameters obtained after the addition of activated protein C (APC) to FVIII-deficient plasmas spiked with emicizumab (50 µg/mL) or aPCC (1.3 IU/mL) are shown. TGA parameters in the presence of APC were compared with those in the absence of APC. Significant differences were considered as p <0.05. One sample in each subgroup was tested per experiment. Experiments were performed three times, and the average values and standard deviation (SD) are shown. The peak thrombin (PeakTh) and endogenous thrombin potential (ETP) values obtained from 15 healthy individuals were 312±76 nM and 2,805±231 nM*min, respectively as described in Methods. PeakTh; peak thrombin, ETP; endogenous thrombin potential. The figures in parenthesis (4), (8), (16) indicate; +APC 4 nM, +APC 8 nM, +APC 16 nM, respectively.

|  | PeakTh | ETP |  |  | PeakTh | ETP |
| --- | --- | --- | --- | --- | --- | --- |
|  | *nM* | *nM×min* |  |  | *nM* | *nM×min* |
| Emi | 199 ± 13 | 3,574 ± 452 |  | aPCC | 297 ± 16 | 4,329 ± 213 |
| Emi (4) | 159 ± 30 | 3,116 ± 519 |  | aPCC (4) | 263 ± 23 | 3,936 ± 729 |
| Emi (8) | 146 ± 28 | 2,892 ± 159 |  | aPCC (8) | 254 ± 21 | 3,736 ± 441 |
| Emi (16) | 117 ± 17* | 2,114 ± 180* |  | aPCC (16) | 242 ± 24 | 3,774 ± 398 |

* p <0.05 *vs* no APC

**Supplementary data**

**Supplementary Figure S1. Impact of emicizumab and FVIII-bypassing agents on AT-induced down regulation in pooled normal plasma (PNP) or FVIII-deficient plasma.**

TF-triggered thrombin generation was assessed after the addition of various concentrations of AT (0-2.4 µM) to PNP or FVIII-deficient plasma supplemented with emicizumab (50 µg/mL) alone, emicizumab and bypassing agents (rFVIIa 2.2 µg/mL, aPCC 1.3 IU/mL, pd-FVIIa/FX 1.5 µg/mL). Experiments were performed three times and the representative AT dose-dependent thrombin generation curves are shown (*blue*; no AT, *red*; 1.2 µM AT, *gray*; 2.4 µM AT).

**Supplementary Figure S2. Effects of APC in pooled normal plasma (PNP) or FVIII-deficient plasma spiked with emicizumab and FVIII-bypassing agents**

TF-triggered thrombin generation in the presence of various concentrations of APC (0–16 nM) was examined in PNP and FVIII-deficient plasmas supplemented with emicizumab (50 µg/mL) and bypassing agents (rFVIIa 2.2 µg/mL, aPCC 1.3 IU/mL, pd-FVIIa/FX 1.5 µg/mL). Experiments were performed three times and representative APC dose-dependent thrombin generation curves are shown (*blue*; no APC, *red*; APC 4 nM, *green*; APC 8 nM, *purple*; APC 16 nM).

**Supplementary Figure S3. APC-induced downregulation of thrombin generation in FVIII-deficient plasma spiked with emicizumab and low dose of aPCC**

TF-triggered thrombin generation after the addition of various concentrations of APC (0–16 nM) was examined in FVIII-deficient plasmas supplemented with emicizumab (50 µg/mL) and aPCC (0.26 or 0.65 IU/mL). Experiments were performed three times and representative APC dose-dependent thrombin generation curves are shown (*blue*; no APC, *red*; APC 4 nM, *green*; APC 8 nM, *purple*; APC 16 nM).

**Supplementary Figure S4. APC-induced downregulation of thrombin generation in FVIII-deficient plasma spiked with emicizumab or aPCC**

TF-triggered thrombin generation was assessed after the addition of various concentrations of APC (0–16 nM) to FVIII-deficient plasmas supplemented with emicizumab (50 µg/mL) or aPCC (1.3 IU/mL). Experiments were performed three times and representative APC dose-dependent thrombin generation curves are shown (*blue*; no APC, *red*; APC 4 nM, *green*; APC 8 nM, *purple*; APC 16 nM).

**Supplementary Figure S5. APC-induced downregulation of thrombin generation in FVIII-deficient plasma spiked with emicizumab and FII or FIXa**

TF-triggered thrombin generation after the addition of various concentrations of APC (0–16 nM) was examined in FVIII-deficient plasmas supplemented with emicizumab (50 µg/mL) and FII (1.3 µM) or FIXa (140 or 280 pM). Experiments were performed three times and representative APC dose-dependent thrombin generation curves are shown (*blue*; no APC, *red*; APC 4 nM, *green*; APC 8 nM, *purple*; APC 16 nM).

**Supplementary Figure S6. APC-mediated downregulation of thrombin generation in FVIII-deficient plasma spiked with emicizumab, FIXa, and rFVIIa or pd-FVIIa/FX or FII**

TF-triggered thrombin generation after the addition of various concentrations of APC (0-16 nM) was examined in FVIII-deficient plasmas supplemented with emicizumab (50 µg/mL), FIXa (280 pM), and rFVIIa (2.2 µg/mL) or pd-FVIIa/FX (1.5 µg/mL) or FII (1.3 µM). Experiments were performed three times and representative APC dose-dependent thrombin generation curves are shown (*blue*; no APC, *red*; APC 4 nM, *green*; APC 8 nM, *purple*; APC 16 nM).
